# Evaluating the interchangeability of infrared and digital devices with the traditional mercury thermometer in hospitalized pediatric patients: an observational study

**DOI:** 10.1038/s41598-021-96587-y

**Published:** 2021-08-23

**Authors:** Angelo Dante, Elona Gaxhja, Vittorio Masotta, Carmen La Cerra, Valeria Caponnetto, Cristina Petrucci, Loreto Lancia

**Affiliations:** Department of Life, Health & Environmental Sciences, University of L’Aquila, Rita Levi Montalcini Building - G. Petrini Street, 67010 L’Aquila, Italy

**Keywords:** Paediatrics, Paediatric research

## Abstract

Gradual replacement of the mercury thermometers with alternative devices is ongoing around the world in a bid to protect human health and the environment from the adverse effects of mercury. However, to reduce the risks of misdiagnosis, unnecessary treatments, and omission of care in pediatric populations, more evidence on the reliability of alternative thermometers is needed. The aim of this comparative observational study was to detect any differences in temperature measurements between the use of the axillary mercury thermometer and the alternative techniques. Temperature values in degree Celsius (°C) were measured in a group of Albanian children aged up to 14 years using mercury and digital axillary thermometers, as well as forehead and tympanic infrared thermometers. The digital axillary device, compared with the mercury one, showed no clinically significant difference in the mean values (− 0.04 ± 0.29 °C) and the narrowest 95% level of agreement (+ 0.53 °C to − 0.62 °C) in the paired comparisons. For cut-off point of 37.5 °C, the digital axillary thermometer showed the highest levels of sensitivity (72.5%) and specificity (99.1%) in detecting fever. This study indicates that the digital axillary thermometer may be the better option since it adequately balances accuracy, safety, and children’s comfort.

## Introduction

Body temperature measurement is an essential component of pediatric health assessment in hospital settings and elsewhere. Normal body temperature values range from 36.5 to 37.4 °C depending on physiological variations, patient characteristics, and sites of measurement^1^.

Since body temperature values, when associated with clinical assessment, contribute to orient diagnoses and therapies for children, unreliable measurements may lead to misdiagnosis, omission or delay of necessary treatments, and prescription of unnecessary therapies or exams^2,3^. For these reasons, body temperature measurement should be carried out with valid and reliable devices^4^.

In this regard, intra-corporeal thermometry methods used to obtain ‘core’ temperature, such as thermistor probes inserted in the pulmonary artery or esophagus, are considered the gold-standard for body temperature measurement^5^. However, these methods are invasive and expensive, could expose patients to risk of complications, and are generally used in critically ill patients^6,7^.

Historically, rectal mercury thermometers were accepted as the gold-standard devices for body temperature measurement in daily clinical practice, but since they cause problems of discomfort and acceptability, the mercury axillaries devices have been used routinely everywhere around the world^5,8,9^.

Some evidence demonstrates that axillary mercury thermometer measurements are only the ‘proxy’ of core body temperature values since they underestimate the internal body temperature by about 1.0 °C. However, the lack of accurate alternative devices, patients’ comfort, and ease of use made this device essential for clinical practice^11^.

The risk posed to the environment and public health by mercury pollution from anthropogenic emissions in air and water has made mercury a global concern and led to governments adopting strategies to reduce its emissions in the atmosphere, soil, and water^10^. In this regard, a series of initiatives aimed at banning the production, import, and export of mercury products, as well as controlling manufacturing processes in which this substance is used, have been carried out, and a gradual replacement of mercury thermometers with alternative devices is ongoing in the health systems of countries who are signatories to the Minamata Convention^10^.

Among the alternative devices, Galinstan-in-glass, digital, and infrared thermometers are currently available in the market; they are easy to use, cost-effective, non-invasive, and safe^11–13^. Nevertheless, only digital axillary and infrared tympanic thermometers are currently recommended in pediatric clinical practice^2,3,14^, since the lack of data confirming the accuracy of other devices in fever diagnosis does not allow them to be considered useful tools for body temperature measurement in pediatric patients^6,7^. However, the guidelines and recommendations for body temperature measurement of pediatric patients are based on moderate quality evidence, and most of the available studies aimed to explore the reliability of the alternative devices as ‘proxy’ measures of core body temperature, instead of exploring their interchangeability with the axillary mercury thermometer^2,8,15,16^.

Therefore, to increase the available evidence and thereby reduce the risks of misdiagnosis, unnecessary treatments, and omission of care in pediatric populations, research on the reliability and interchangeability of the alternative devices in clinical practice is ongoing globally^4,8,13,17–20^. It is in this regard that we undertook this study, with the aim to detect any differences in body temperature measurements obtained with the axillary mercury thermometer and those obtained with the new digital and infrared devices in a pediatric setting. The study hypothesis was that no clinically significant differences existed between the old mercury thermometer and the new devices, especially in regard to sensitivity and specificity, for fever detection.

## Methods

### Study design, setting, and participants

A comparative observational study was conducted from September 2018 to January 2019 in a fifty-bed pediatric ward of a general hospital in Albania, where about one thousand patients are admitted annually for a broad spectrum of medical health issues ranging from respiratory diseases to infectious diseases such as enteritis. Albania is one of the developing countries of Europe, where the use of mercury thermometers was still allowed when the study was conducted.

Using consecutive sampling, all pediatric patients aged up to 14 years and requiring body temperature measurements were enrolled if the parents gave their informed consent. Hospitalized children in critical conditions or those unable to tolerate multiple body temperature measurements were excluded. Referring to subjects that had to receive body temperature measurements twice a day, a sample size of at least 327 children was estimated to provide a 95% power (1 − β) and a 5% α error in detecting body temperature measurement differences, using G* Power 3.1.9.2 software.

### Variables

To perform a secondary analysis of subgroups’ potentially affecting differences in body temperature detection between the compared thermometers, data on demographic and clinical variables, such as age, gender, site of body temperature measurements, admission diagnosis, and antipyretic drug administration, were also collected. In this study, the axillary mercury thermometer was considered the reference standard while digital axillary, infrared forehead, and tympanic devices were the alternative measurement methods.

### Instruments and data collection

Following each thermometer manufacturer’s instructions, body temperature measurements were collected, twice a day, at 8:00 in the morning and 5:00 in the afternoon, using the investigated devices in this sequence: axillary mercury, digital axillary, infrared tympanic, and infrared forehead.

For axillary temperature detection, GEA Medical Mercury thermometers (Product code 10901902464, GEA®, Indonesia) and Easy Touch Digital thermometers (Product code 00006929000000, Chicco®, Italy) were used, whereas for tympanic and forehead temperature detection Infra-Red Comfort Quick devices (Product code 00000656000000, Chicco®, Italy) and Infra-Red Easy Touch thermometers (Product code 00004757100000, Chicco®, Italy) were respectively used. The manufacturer of the alternative thermometers reported a ± 0.1 °C measurement error for the digital axillary device (body temperature range from 35.5 to 42.0 °C) and a ± 0.2 °C error for both the infrared tympanic and forehead thermometers (body temperature range from 35.0 to 42.0 °C).

Both the axillary devices were placed deeply in each child’s left or right armpit with the arm held steady; it took one and five minutes to measure body temperature with the axillary digital and axillary mercury devices, respectively. The mercury thermometer was used after making sure the mercury level had gone down to the minimum (35.0 °C). Recordings were timed through a chronometer for the mercury device and its beeper for the digital device.

The tympanic temperature was detected by scanning the infrared radiation from the tympanic membrane for one second. For each measurement, the probe of the tympanic thermometer was replaced, and taking measurements in the ear in contact with a pillow was avoided.

As recommended by the manufacturer, the forehead temperature was measured by scanning the infrared radiation from the temporal artery for about five to eight seconds (maximum 30). Using the same side for temporal measurements in the same child prevented intraindividual body temperature differences due to blood vessel depth.

All measurements were performed on clean and dry skin, waiting at least 30 minutes after meals or baths. Prior to their use, thermometers were set according to the manufacturer’s instructions, if required.

To ensure the accuracy of measurements, five Albanian nurses, having attended a theoretical–practical training about the characteristics and usage of new thermometers and about the research protocol, performed all body temperature measurements and recorded the data on a body temperature flowsheet under the supervision of one of the researchers.

### Data analysis

Data were summarized using frequencies (n), percentages (%), central tendency indexes (mean and median), and dispersion measures, such as standard deviation (SD), interquartile range (IQR), and range. After the non-normal distribution of continuous data was graphically assessed using histograms, boxplots, and Q–Q plots, and verified with the Kolmogorov–Smirnov test, differences between body temperature values obtained through the mercury thermometer and the alternative devices were statistically checked using the Wilcoxon test and visually compared using the Bland–Altman scatterplots^21,22^. Considering as undesirable the differences between the axillary mercury thermometer and other devices’ measurements, a maximum significant level of 0.05 was considered for the Wilcoxon test, whereas for the Bland–Altman analysis 95% Limits of Agreement (LoA), defining the range within which most body temperature differences fell, were computed with the formula ‘*mean of body temperature measurement differences* ± 1.96 × SD’^23,24^; mean values of ± 0.5 °C were considered the maximum acceptable LoA based on the available evidence^6^. The statistical difference of the proportions of mean differences between the mercury thermometer and other devices, which fell under the ± 0.5 °C maximum acceptable LoA, was tested by the Chi-square test.

Finally, to analyze the diagnostic accuracy of the alternative devices in detecting fever, their sensitivity ‘true positives’/(‘true positives’ + ‘false negatives’) and specificity ‘true negatives’/(‘true negatives’ + ‘false positives’) were calculated^24^. For this purpose, a cut-off to discriminate fever/no-fever conditions needed to be fixed, and since normal body temperature values are related to the site of measurement and no international agreement has been reached on the exact values to define fever^1,25,26^, this study considered the peripheral body temperature ≥ 37.5 °C as cut-off value to include febrile patients^1,27,28^.

All data were analyzed using IBM SPSS version 25.0 (IBM Corp., Armonk, New York, USA).

### Ethics

This study is the result of an international cooperation between Italian and Albanian academic institutions and was conducted in accordance with the Declaration of Helsinki. The study was approved by the Institutional Review Board of the Hospital of Elbasan, Albania (letter of approval #1693/2018). Before data collection, the study aims were explained to the children’s parents and their written informed consent was obtained in Albanian language. Albanian nurses who performed data collection as well as the local manager were available to clarify doubts and answer any question related to the study. Nevertheless, no child was forced to participate if there was verbal or non-verbal refusal.

## Results

### Participants

A total of 356 pediatric patients were enrolled (Table 1). Two hundred and eleven (59.3%) were male and the median age was 2.0 years (range 0–14). The main reasons for hospitalization were respiratory (209, 59.3%) and gastrointestinal diseases (73, 20.6%). Forty patients (11.2%) received antipyretic drug administration before body temperature measurements.

**Table 1:** Participants' characteristics (n = 356).

	n	%
**Gender**		
Male	211	59.3
Female	145	40.7
**Age**		
Mean ± (SD)	3.0 ± (3.0)	
Median, (IQR)	2.0 (3.1)	
Admission diagnosis (grouped by system)*		
Respiratory	209	59.0
Gastrointestinal	73	20.6
Neurologic and sensorial	29	8.2
Urinary	9	2.5
Locomotor and articular	3	0.8
Other	31	8.8
**Antipyretic drugs administered before BT measurements**		
Yes	40	11.2
No	316	88.8

*Missing data n = 3.

### Differences in body temperature measurements between the alternative devices and the axillary mercury thermometer

**Figure 1:**
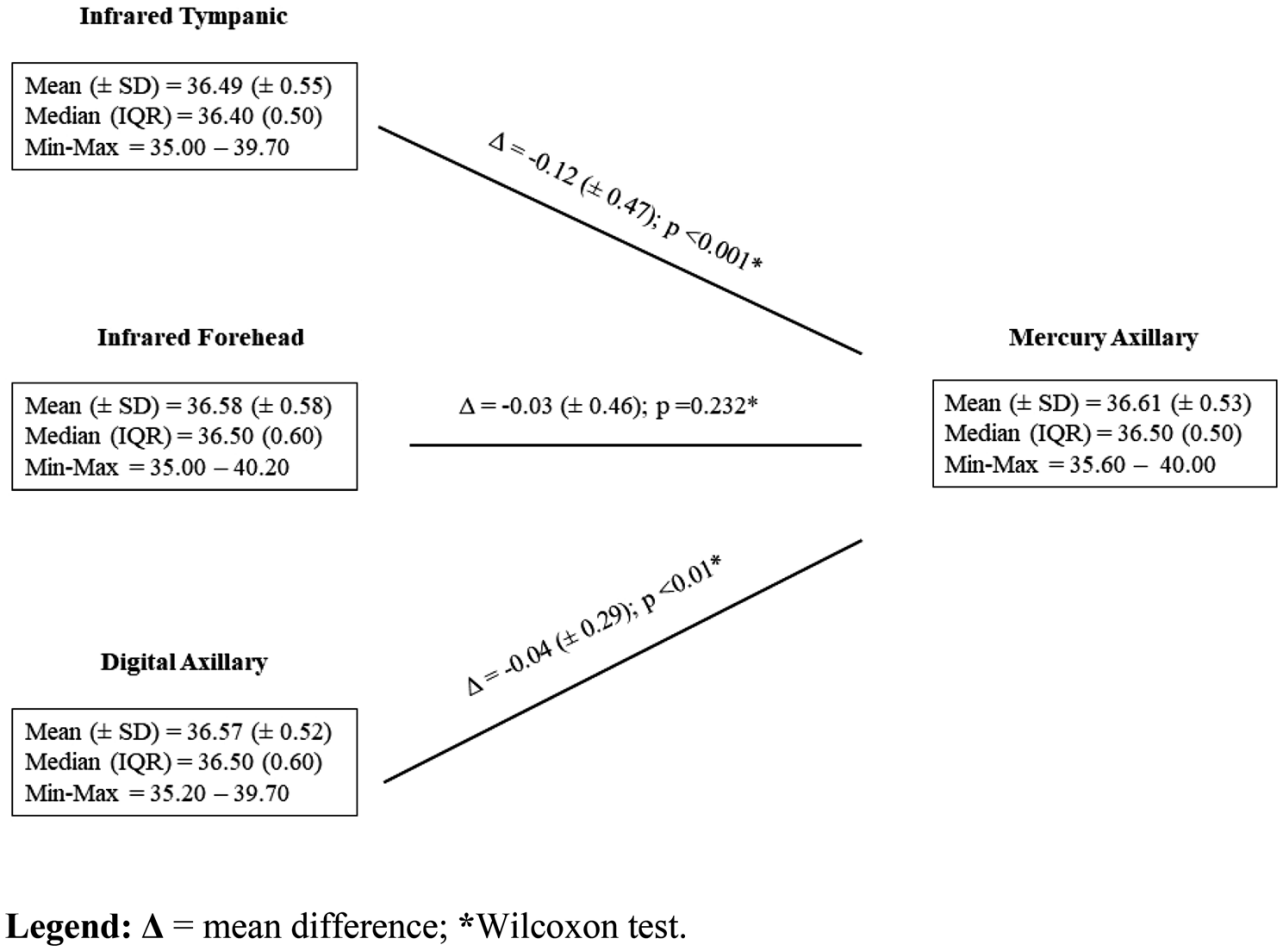
Paired comparisons between the axillary mercury thermometer and the alternative ones (Measurements = 711). Δ = mean difference; *Wilcoxon test.

As shown in Fig. 1, in a paired comparison with the axillary mercury device, both the digital axillary and infrared tympanic devices showed slightly lower mean body temperature values, which were 0.04 (median 0.00; IQR 0.3; p < 0.001) and 0.12 °C (median − 0.10; IQR 0.6; p < 0.001), lower than that given by the axillary mercury device, respectively, while the mean differences between the body temperature values given by the reference standard and the infrared forehead thermometer (0.03 °C; median 0.00; IQR 0.5) were not statistically significant (p = 0.232).

**Figure 2:**
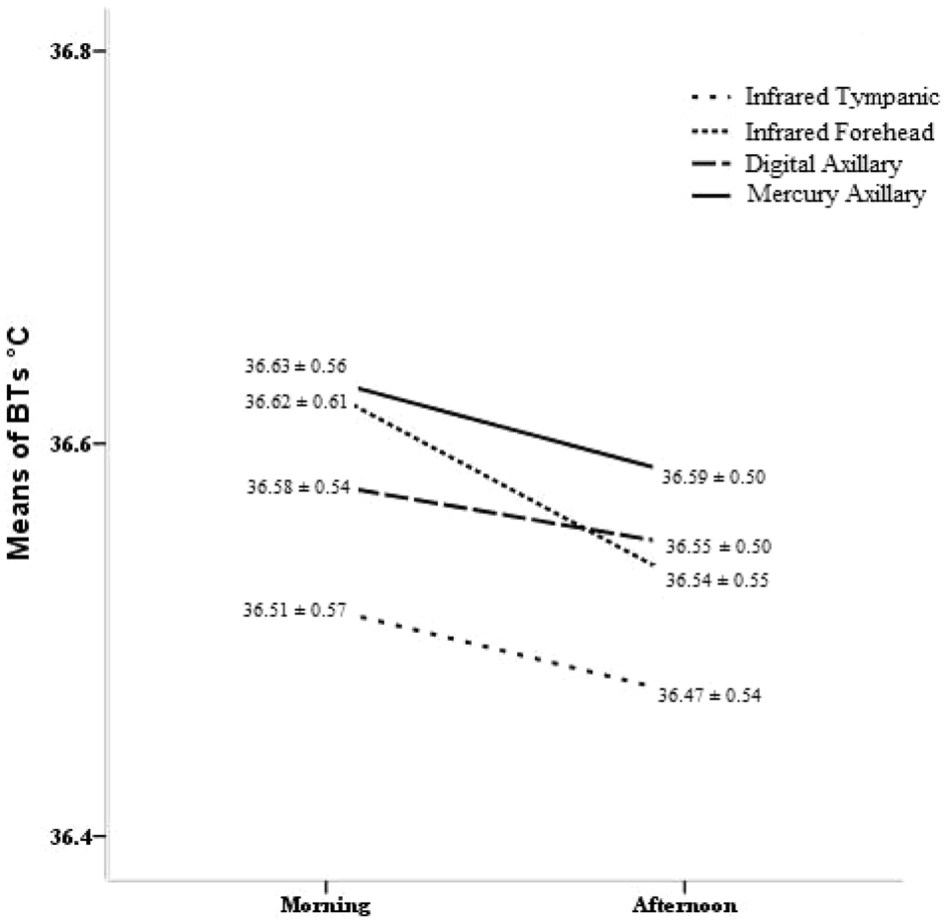
Body temperature values and time of the day.

With reference to daily body temperature variations, the average data of the measurements taken in the morning and afternoon showed that all the alternative thermometers reported values less than that reported by the mercury thermometer (Fig. 2), although all the body temperature variations fell within the maximum measurement error provided by the manufacturer for each device.

**Figure 3:**
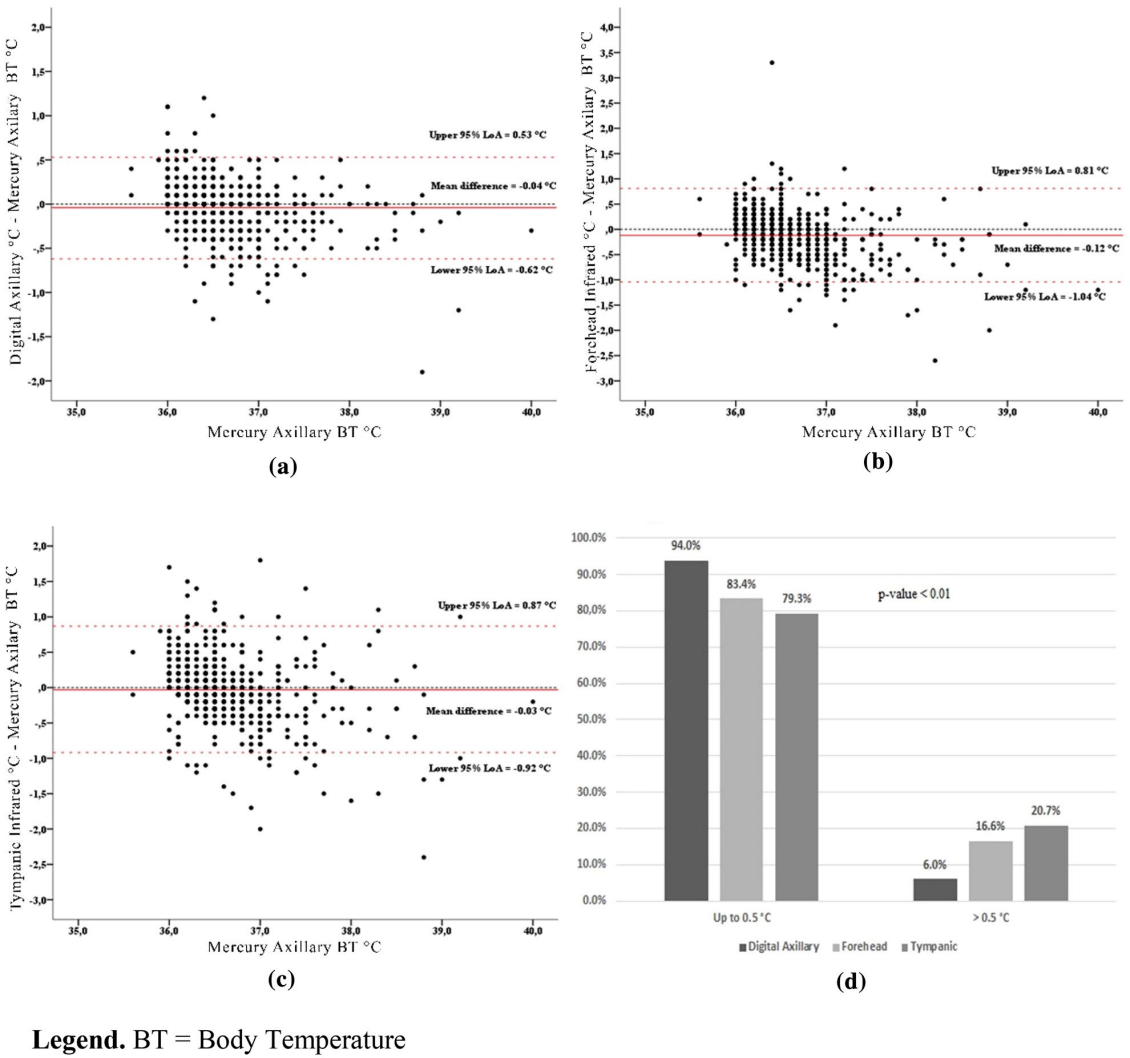
Paired comparisons between the axillary mercury thermometer and the other devices. *BT* Body Temperature; (**a**) Digital Axillary vs. Mercury Axillary; (**b**) Forehead Infrared vs. Mercury Axillary; (**c**) Tympanic Infrared vs. Mercury Axillary; (**d**) Percentage of mean differences between Mercury and other thermometers falling under 0.5 °C.

The Bland–Altman scatterplots (Fig. 3a–c) showed the narrowest 95% LoA (+ 0.53 °C to − 0.62 °C) for the measurement comparison between the axillary mercury thermometer and the digital axillary device, and the broadest 95% LoA (+ 0.81 °C to − 1.04 °C) for the comparison between the axillary mercury and infrared tympanic values. In all the Bland–Altman scatterplots, the magnitude of differences between the alternative and mercury thermometers decreased when the average body temperature values increased.

Taking ± 0.5 °C as the maximum clinically acceptable difference, when the three alternative devices were compared to the reference standard, 94.0%, 83.4% and 79.3% of values detected with the digital axillary, infrared forehead and tympanic thermometers, respectively, fell within these cut-offs (Fig. 3d). These differences were statistically significant (p < 0.001).

In addition, across the entire sample, the digital axillary thermometer showed the highest levels of sensitivity and specificity in detecting fever, regardless of the established cut-off (Table 2).

**Table 2:** Diagnostic accuracy of alternative devices to detect fever compared to mercury thermometer (measurements = 711).

Indexes	AXL_DGT_	FHD_IR_	TYM_IR_
**(BT ≥ 37.5 °C)**			
Sensitivity	72.5	64.7	51.0
Specificity	99.1	96.8	98.3

## Discussion

This study aimed at detecting any differences in body temperature measurements between the use of the axillary mercury thermometer, and the use of the new digital and infrared thermometers in a pediatric hospital setting in Albania, where mercury thermometers were still allowed despite the Minamata Convention, due to a transition phase^10^. On 26th May 2020, the Government of Albania deposited its instrument of ratification, becoming the 120th Party to the Minamata Convention.

To replace mercury thermometers, numerous environment-friendly devices have been introduced in clinical settings all around the world in the last 10 years, and a great amount of research has been conducted to explore their reliability^11–13^. Some recent meta-analyses carried out to compare peripheral electronic devices (e.g. digital axillary, infrared forehead, and tympanic) with central devices (e.g. bladder, esophageal and rectal) indicated that the peripheral devices showed poor accuracy when used to estimate core body temperature and inadequate sensitivity when used for fever detection in adults and children^6,7,16^. In particular, the forehead thermometer was not found sufficiently accurate to replace one of the investigated reference methods, such as rectal, bladder, nasopharyngeal, esophageal, and pulmonary, both in adult and in children^6^. Other authors, comparing the forehead thermometer with the rectal, nasopharyngeal, esophageal, and urinary bladder devices in pediatric patients, highlighted a low sensitivity for detecting fever and recommended caution in its use in clinical practice^7^. Finally, the peripheral electronic devices (digital axillary, tympanic, and forehead) compared with the esophageal, pulmonary, urinary bladder, and rectal thermometers showed no clinically acceptable level of agreement; for this reason, they are not recommended in clinical practice, with the exception of the tympanic device^16^.

However, using a different research methodology, namely, comparing the peripheral thermometers among themselves without reference to core body temperature, achieved contradictory evidence^29–32^. For example, some studies found that the infrared forehead thermometer could serve as a good alternative to the digital axillary device due to its user-friendliness and speed of use^33^, while others, reporting great mean differences between the investigated devices, did not consider the forehead device as accurate as the digital axillary thermometer^19,31,33^.

The research approaches in the above-mentioned studies had two distinct goals: comparing the peripheral devices with the central ones and exploring the level of agreement among the alternative thermometers. Surprisingly, available evidence has not completely dissolved nurses’ concerns about the reliability of the new thermometers. In fact, when they need to make clinical decisions aimed, for example, at improving the child’s comfort, reducing parental anxiety, minimizing environmental influences on thermoregulation, preventing dehydration, assessing the signs and symptoms for serious illnesses or infections, and consulting a pediatrician for the prescription of drugs or additional tests, they need to make sure that the digital and infrared devices are at least as reliable as the old axillary mercury thermometer they used for a long time. In the light of our results, the research hypothesis of no clinically significant differences between the old axillary mercury thermometer and the new devices should be partially rejected. In fact, significant mean differences were found for the paired body temperature comparisons between each of the digital axillary and infrared tympanic devices and the mercury thermometer (− 0.04 °C and − 0.12 °C, respectively). However, in this case, statistically significant differences cannot be said to correspond to strong clinical significance since it is unlikely that a maximum difference of − 0.12 °C can affect some clinical judgements, such as drug administration or caring interventions. Beyond the statistical significance, in the visual analysis of differences between measurements (Bland–Altman scatterplots), it can be seen that the digital axillary values are the closest to the axillary mercury, since 95% of their differences fell within the narrowest range (95% LoA = − 0.62 °C to + 0.53 °C). In addition, the digital axillary thermometer showed the highest percentage (94.0%) of body temperature differences within the clinically acceptable value of ± 0.5 °C^6^. These results are not surprising, since both the digital and mercury axillary thermometers were used to detect body temperature in the same body site, and site of measurement is one of the most relevant factors that affect body temperature values^1^. Furthermore, the digital axillary thermometer showed better performance in screening for fever than infrared devices, even if in this study all the investigated devices showed moderate to low sensitivity and high specificity in detecting fever. Also, the digital axillary thermometer, to a lesser extent than the other alternative devices, resulted in a higher proportion of false-negative than false-positive readings, which could be explained by the tendency in this study to underestimate the axillary mercury measurements. In practice, using the digital axillary thermometer in a minimal proportion of children found to be non-febrile, fever could not be ruled out with certainty. Unfortunately, the sensitivities and specificities of the alternative devices in detecting fever were not estimated using core body temperature (esophageal, vesical, pulmonary) or the clinically acceptable gold standard (rectal) measurement methods^6,7,16,27^. For this reason, even if the results of this study seem to suggest that the digital axillary device may be preferable for body temperature measurement in children, it is advisable, in accordance with current guidelines, that a conservative approach, e.g. the use of rectal thermometers, is used to confirm fever, in order to protect children from missed care, especially when clinical signs and symptoms contrast with detected body temperature values^2,3,14^.

An innovative aspect of this study is that, in all the Bland–Altman scatterplots, the magnitude of differences between the alternative and mercury thermometers decreased when the average body temperature values increased. This implies that in cases of high body temperature that deserve more clinical attention, the agreement of the alternative devices with the reference standard improves. Even if this could have possible clinical implications related to the use of thermometers, the low number of febrile children investigated in this study will not allow us to draw this conclusion. Further studies are needed to confirm this data.

### Practice implications

According to current guidelines^2,3,14^, the results of this study suggest that the digital axillary thermometer may be the best choice for body temperature measurement in pediatric settings, considering especially that this device adequately balances accuracy, safety, and children’s comfort. Even if there are practical reasons that seem to favor the use of the infrared tympanic and infrared forehead thermometers in pediatric clinical practice, such as their ease of use, speed of measurements and improved hygiene, the results of this study show that these devices cannot be considered interchangeable with the mercury thermometer, and the digital axillary device should be preferred. However, considering the performance of the digital axillary thermometer in the screening of fever, clinical decisions should not be based exclusively on body temperature values, but, in accordance with current guidelines, it is always advisable to assess children for the presence or absence of signs and symptoms potentially associable with fever^3^. Assessing the skin color and turgor, respiratory function, cardio-circulatory condition, the child’s activity, and the presence of headache, shiver, and nausea provides excellent criteria to confirm or disprove a body temperature value^3,14^. This last recommendation should be strongly considered for clinical practice especially during epidemic events, such as the current Coronavirus disease 2019 (COVID-19) pandemic. In this regard, one of the special accommodations made in clinical practice and other contexts is for the use of the infrared thermometers. For its ‘no contact’ process which limits the virus spread, the infrared forehead device has become the most widely accepted thermometer in this pandemic. However, considering that in this study fever (≥ 37.5 °C) in about one-third of febrile children could not be detected with the infrared forehead device, temperature screening alone should be avoided in every context since it may not be very effective, as previous evidence has supported^7^. The signs and symptoms commonly present in children with the Coronavirus disease 2019, such as fatigue, dry cough, and other respiratory symptoms, should be considered along with body temperature values^34,35^.

### Limitations of the study

The strengths of this study included the adequate sample size of pediatric patients, the measurement of body temperature in a real clinical setting, and the use of appropriate statistical methods for data analysis. However, the results of this study should be accepted bearing in mind its monocentric approach and the differences between core and peripheral body temperature values. In this regard, the use of the axillary mercury thermometer as a reference standard instead of core body temperature detection methods (pulmonary, esophageal, or intra-vesical) represents a limitation of this study. However, it allowed us to evaluate the interchangeability of the new digital and infrared devices with the axillary mercury thermometer historically used in clinical practice, although the temperature values given by these devices are only a proxy of the true core body temperature. Finally, the intrinsic differences in body temperature related to different sites of measurements should be considered while interpreting the level of agreement between the compared devices.

## Conclusion

The results of this study confirm the digital axillary device as the best alternative to the axillary mercury thermometer in detecting children’s body temperature both in cases of fever or not. However, according to current guidelines, when clinical signs and symptoms contrast with detected body temperature values, it is recommended that the body temperature measurements be repeated or rectal thermometers be used.

## Data Availability

Data and materials are available from the corresponding author on reasonable request.
